# Supplementary material for: CRISPR-Cas9-assisted genome editing in E. coli elevates the frequency of unintended mutations
Source: bioRxiv. 2024 Mar 19:2024.03.19.584922. Preprint. [Version 1] doi: 10.1101/2024.03.19.584922 (PMC10983943; doi:10.1101/2024.03.19.584922)
Supplement: Supplement 3 [file media-3.pdf]

Supplemental Figures

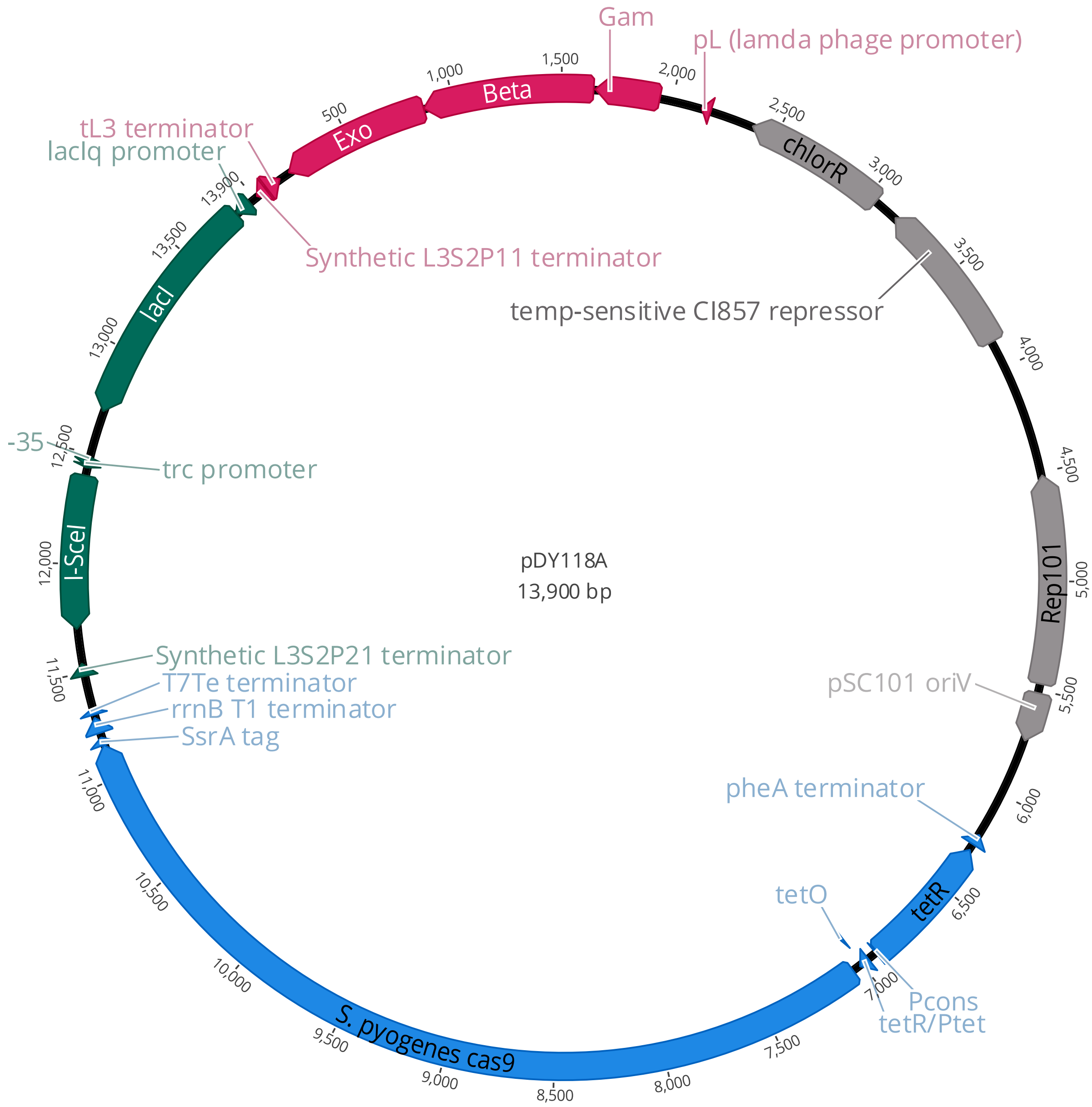

Figure S1. Helper plasmid pDY118A. Blue, SsrA-tagged *S. pyogens* Cas9 under regulation of an anhydrotetracycline-inducible promoter; green, I-SceI under regulation of an IPTG-inducible promoter; red, lambda Red proteins Exo, Beta and Gam under regulation of a heat-inducible promoter. pSC101 temperature-sensitive origin of replication allows plasmid to be maintained at 30 °C and cured at 37 °C.

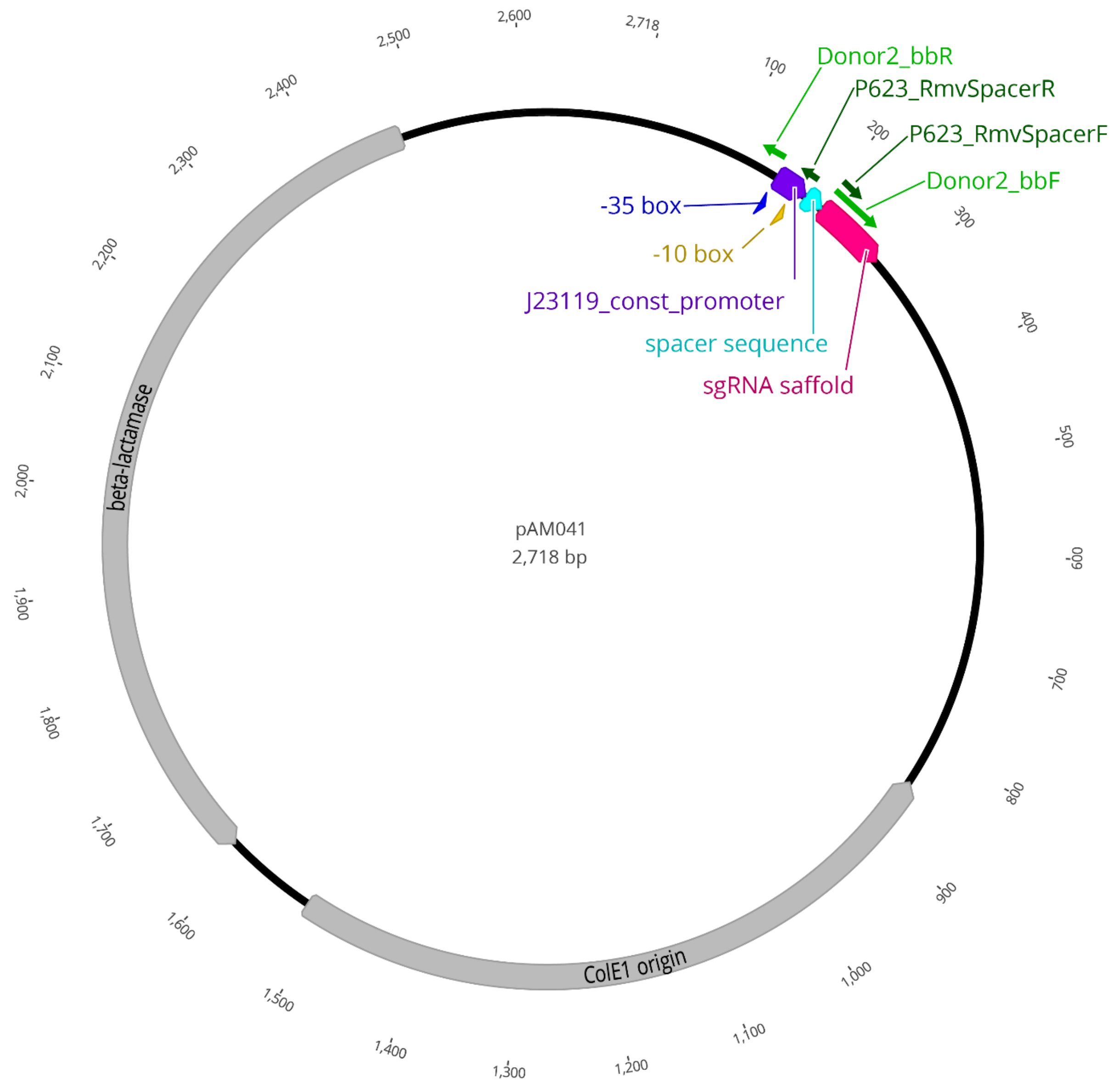

Figure S2. pAM041, the plasmid from which backbones for donor and guide plasmids were amplified. Constructed from pSS9_gRNA (1). Purple, promoter for sgRNA; cyan, 20-nucleotide spacer sequence targeting sequences; red, sgRNA scaffold; dark and light green, primer pairs used to amplify the backbone for guide and donor plasmids, respectively. Insertion site for editing cassettes used in donor plasmids is immediately upstream of the promoter.

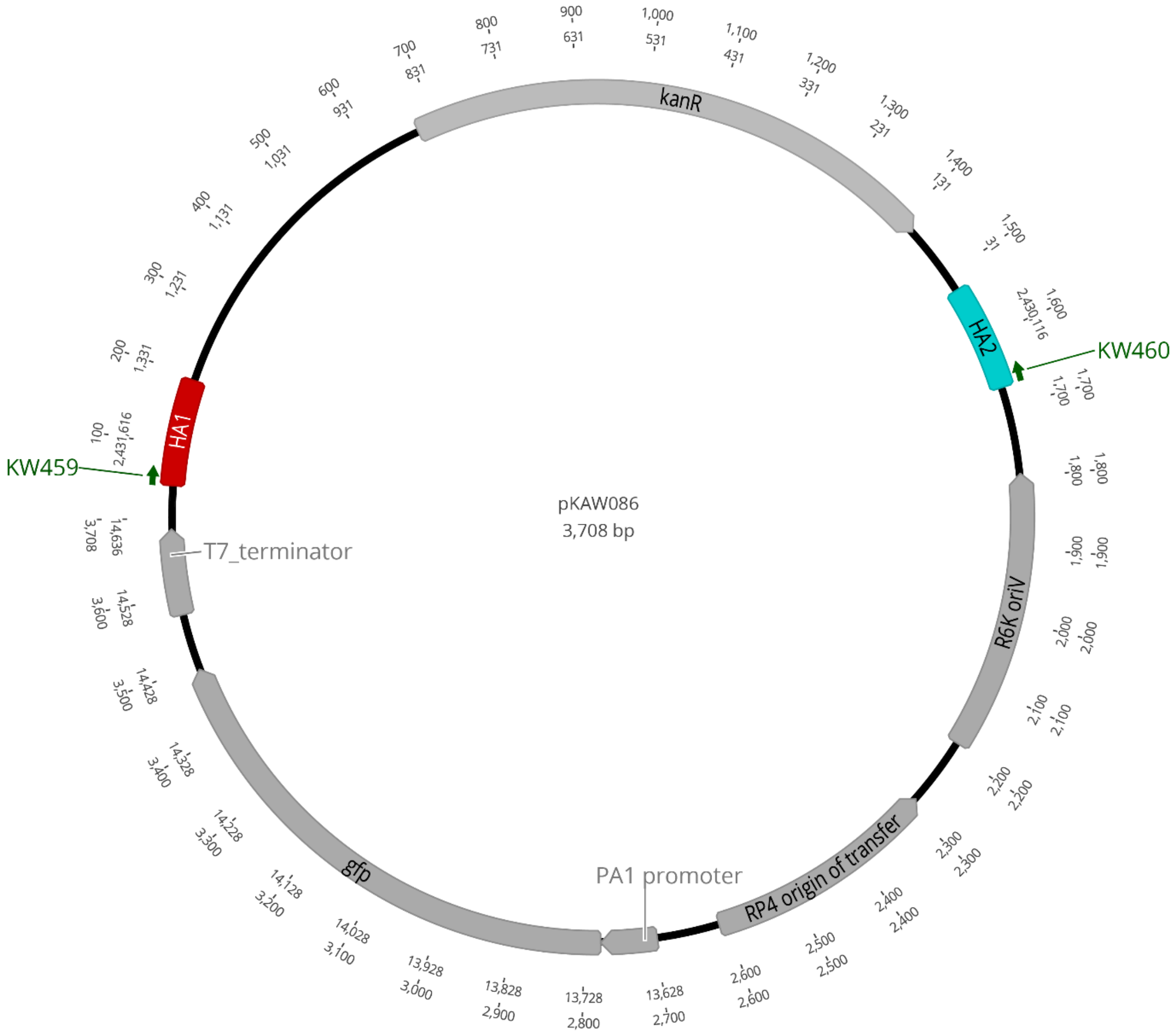

Figure S3. pKAW086, which contains the editing cassette used in lambda Red recombineering experiments. Red and cyan, regions with homology upstream and downstream of *E. coli pdxB,* respectively; dark green, primers used to amplify the editing cassette.

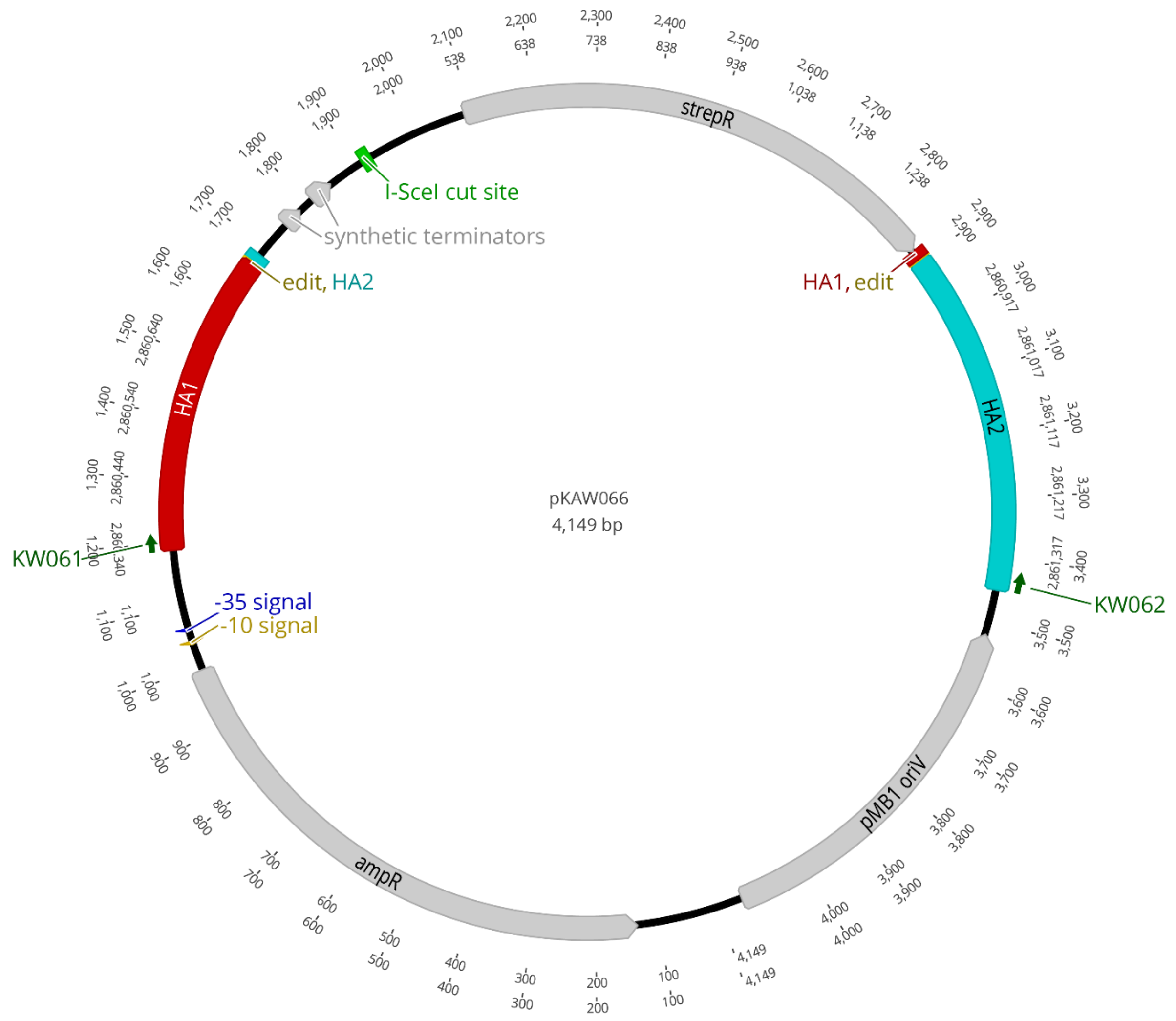

Figure S4. pKAW066, which contains the editing cassette used in I-SceI and is comprised of three segments: first, a segment containing a homology arm (HA) upstream of the edit (large red HA1), the edit (yellow) and a short downstream HA (small cyan HA2); second, a segment containing two synthetic terminators (the synthetic terminators ensure efficient cleavage at the I-SceI site), an I-SceI cut-site (green) and a streptomycin resistance gene (grey); and third, a segment containing a short HA upstream of the edit (small red HA1), the edit (yellow) and a downstream HA (large blue HA2). Dark green, primers used to amplify the editing cassette.

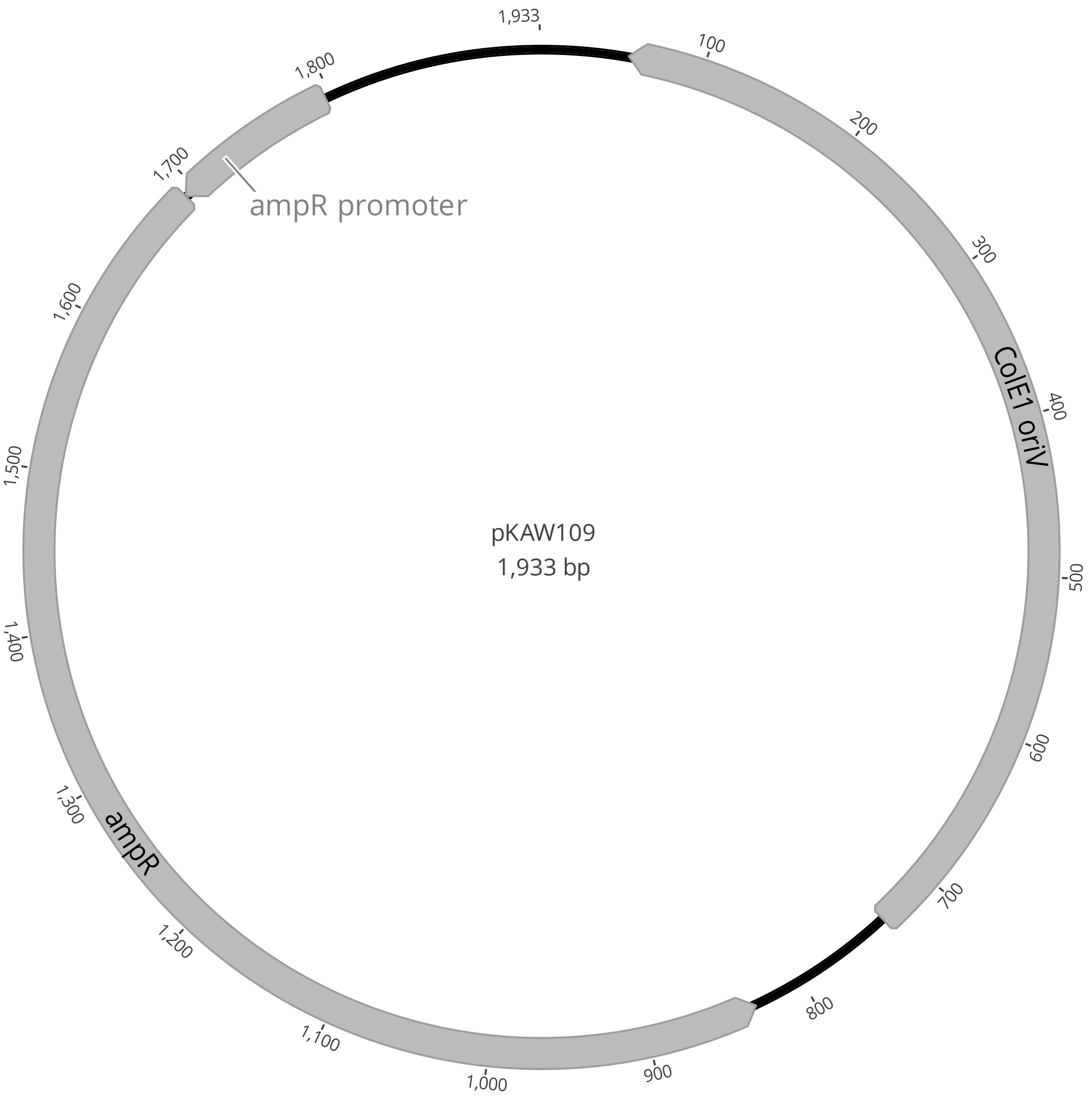

Figure S5. pKAW109, the plasmid used in control experiments. The backbone was amplified from pAM041 (Figure S2) but lacks an editing cassette and sgRNA.

Supplemental Tables

**Table S1.** Strains used in this work. All strains except DH5a and DH5a lambda pir contained the helper plasmid pDY118A. Intm designates intermediate strains that had previously been edited and were used for a second round of genome editing.			
strain	genotype	notes	Biosample accession
E. coli DH5α	*Δ(argF-lac)169*, φ80d*lacZ58*(M15), *ΔphoA8*, *glnX44*(AS), λ^-, *deoR481*, *rfbC1*, *gyrA96*(NalR), *recA1*, *endA1*, *thiE1*, *hsdR17*	Coli Genetic Stock Center (CGCS) strain # 14231	
E. coli DH5α lambda pir	DH5α with genomic integration of gene encoding Pir	obtained from Eric Stabb (2)	
E. coli BW25113*	BW25113 (MG1655 *Δ(araD-araB)567*, *ΔlacZ4787*(::rrnB-3), λ^-, *rph-1*, *Δ(rhaD-rhaB)568*, *hsdR514)*; *pdxB* replaced with *kanR*	parental strain for all I-SceI edited strains	SAMN40533614
E. coli BW25113* *yifE Q100**	BW25113* with a C → T mutation at position 298 in *yifE* resulting in a Q100* missense mutation		SAMN40533615
E. coli BW25113* *gapA**	BW25113* with a G → T mutation at position 629 in *gapA* resulting in a G210V substitution		SAMN40533616
E. coli BW25113* *gapA* ΔacrZ-pgl*	BW25113* with a G → T mutation at position 629 in *gapA* resulting in a G210V substitution and a 3812 bp deletion spanning genomic positions 790371-794183		SAMN40533617
E. coli BW25113* *gapA* ΔacrZ-pgl rpoC**	BW25113* with a G → T mutation at position 629 in *gapA* resulting in a G210V substitution, a 3812 bp deletion spanning genomic positions 790371-794183 and a C → A mutation at position 2788 in *rpoC* resulting in an L930M substitution		SAMN40533618
E. coli MG1655*	MG1655 with 82 bp deletion upstream of *pyrE; gmhB / rrsH* intergenic (+240/-123, A → G); *paoD* E266K (GAG → AAG); *sixA* E61A (GAA → GCA)*; gltP / yjcO* intergenic (+587/+55) +GC	parental strain for all Cas9-assisted editing	SAMN40533619
E. coli MG1655* *acs*328::mCherry intm A	MG1655*, codons 328-331 of *acs* replaced with mCherry-NGG (CTGTCCCCTCAGTTCATGTAagg) ; *chaA*/*chaB* intergenic mutation (-120/-150) A → G	unintended mutation between *chaA* and *chaB* occurred while introducing *acs*328::mCherry	SAMN40533620
E. coli MG1655* *acs*328::mMCherry intm B	MG1655*, codons 328-331 of *acs* replaced with mCherry-NGG	unintended mutation between *frmR* and *yaiO*	SAMN40533621

	(CTGTCCCCTCAGTTCATGTAagg), *frmR*/*yaiO* intergenic 1 bp deletion (-132/+56)	occurred while introducing *acs*328::mCherry	
E. coli MG1655* *acs*328::mCherry intm C	MG1655*, codons 328-331 of *acs* replaced with mCherry-NGG (CTGTCCCCTCAGTTCATGTAagg)		SAMN40533622
E. coli MG1655* *acs*610::mCherry intm	MG1655*, codons 610-624 of *acs* replaced with mCherry-NGG (CTGTCCCCTCAGTTCATGTAagg)		SAMN40533623
E. coli MG1655* NEW_*ackA*105-2::mCherry intm	MG1655*, codons 105-114 of *ackA* replaced with mCherry-NGG (CTGTCCCCTCAGTTCATGTAagg)		SAMN40533624
E. coli MG1655* *adhE*490::mCherry intm A	MG1655*, codons 486-496 of *adhE* replaced with mCherry-NGG (CTGTCCCCTCAGTTCATGTAagg)		SAMN40533625
E. coli MG1655* *adhE*490::mCherry intm B	MG1655*, codons 486-496 of *adhE* replaced with mCherry-NGG (CTGTCCCCTCAGTTCATGTAagg)		SAMN40533626
*E. coli MG1655** *cyaA*499::mMCherry intm	MG1655*, codons 497-500 of *cya* replaced with mCherry-NGG (CTGTCCCCTCAGTTCATGTAagg)		SAMN40533627
E. coli MG1655* *fbaB*236 syn intm	MG1655*, codons 230-237 of *fbaB* replaced with synonymous codons (ACAATAGGAGcggACATTGTGAAG)	mutated codons include a novel ‘cgg’ PAM site.	SAMN40533628
E. coli MG1655* *pgi*352 syn intm	MG1655*, codons 344-352 of *pgi* replaced with synonymous codons (CGGTTTGCAGCCTATTTTCAACAa)	final codon ending with ‘a' is followed by ‘gg’ in the genome, providing a novel ‘agg’ PAM site	SAMN40533629
E. coli MG1655* *ptsI*513 syn intm	MG1655*, codons 509-514 of *ptsI* replaced with synonymous codons (GAGAGAGCAACCTTGTTACTATTa)	final codon ending with ‘a’ is followed by ‘gg’ in the genome, providing a novel ‘agg’ PAM site	SAMN40533630
E. coli MG1655* pykF085/090 syn intm	MG1655*, codons 81-91 of *pykF* replaced with synonymous codons (AATGATGTCAGcctAAAGGCCGGACAAACGTTC)	mutated codons include a reverse complimentary novel ‘agg’ PAM site	SAMN40533631
E. coli MG1655* *pykF*466 syn intm	MG1655* background, codons 451-466 of *pykF* replaced with synonymous codons (ATGGTCAGCGGAGCTTTAGTTCCATCaggAACAACGAATACCGCTAGC)	mutated codons include a reverse complimentary novel ‘agg’ PAM site	SAMN40533632

**Table S2.** Plasmids used in this work.		
plasmid	use / description	source or reference
pDY118A (Fig S1)	helper plasmid for Cas9-assisted editing; encodes Cas9 under control of the Tet promoter, lambda Red enzymes under control of a heat-inducible promoter, I-Sce-I under control of the lacIq promoter, and chloramphenicol resistance; pSC101 temperature-sensitive origin of replication	(3)
pAM041 (Fig S2)	backbone for donor and guide plasmids used for Cas9-assisted editing (Addgene ID 217969)	this study
Cas9 donor plasmids	encode ampicillin resistance, an sgRNA and an editing cassette	
pDY339	sgRNA targets an mCherry-AGG sequence introduced at codon 105 of *ackA* through a previous round of genome editing; repair template introduces three synonymous mutations at codons 105, 113 and 114 (Table S4, GB_ackA105_2nd)	(3)
pDY348	sgRNA targets an mCherry-AGG sequence introduced at codon 499 of *cyaA* through a previous round of genome editing; repair template introduces two synonymous mutations at codons 497 and 498 (Table S4, GB_cyaA499_2nd)	this study
pDY449	sgRNA targets a sequence of synonymous codons introduced at codons 84-92 of *pykF* by a previous round of genome editing; repair template introduces two synonymous mutations at codons 85 and 86 (Table S4, GB_pykF085_2nd)	(3)
pDY450	sgRNA targets a sequence of synonymous codons introduced at codons 84-92 of *pykF* by a previous round of genome editing; repair template introduces five synonymous mutations at codons 83, 84 and 90 (Table S4, GB_pykF090_2nd)	(3)
pDY452	sgRNA targets a sequence of synonymous codons introduced at codons 453-459 of *pykF* by a previous round of genome editing; repair template introduces seven synonymous mutations at codons 452, 453 and 466 (Table S4, GB_pykF466_2nd)	(3)
pDY453	sgRNA targets a sequence of synonymous codons introduced at codons 453-459 of *pykF* by a previous round of genome editing; repair template introduces four synonymous mutations at codons 452 and 453 (Table S4, GB_pykF470_2nd)	(3)
Cas9 guide plasmids (Fig S2)	encode ampicillin resistance and an sgRNA but no editing cassette	
pDY320	sgRNA targets an mCherry-AGG sequence introduced at codon 328 of *acs*, codon 610 of *acs* or codon 490 of *adhE* by previous rounds of genome editing	(3)
pDY332	sgRNA targets a sequence of synonymous codons introduced at codons 344-352 of *pgi* by a previous round of genome editing	(3)
pDY334	sgRNA targets a sequence of synonymous codons introduced at codons 230-237 of *fbaB* by a previous round of genome editing	(3)
pDY337	sgRNA targets a sequence of synonymous codons introduced at codon 509-514 of *ptsI* by a previous round of genome editing	this study
lambda Red recombineering	encodes GFP and contains an editing casette to replace *pdxB* with *kanR*	

mutation cassette plasmid (Fig S3)		
pKAW086	donor plasmid for lambda Red recombineering; editing cassette contains *kan* with homology arms flanking *E. coli pdxB*; encodes GFP under control of a constitutive promoter; R6K origin of replication that requires Pir to be maintained in cells; kanamycin and ampicillin resistance (Addgene ID 217967)	this study
I-SceI mutation cassette plasmid (Fig S4)	editing cassette used to introduce a point mutation in *rpoS* via I-SceI-assisted editing; ampicillin resistance	
pKAW066	donor plasmid for I-SceI assisted editing; editing cassette comprised of three segments: first, a segment containing a homology arm (HA) upstream of the edit, the edit and a short downstream HA; second, a segment containing an I-SceI cut-site and a streptomycin resistance gene; and third, a final segment containing a short HA upstream of the edit, the edit and a downstream HA; ampicillin resistance; pMB1 origin of replication (Addgene ID 217968)	this study
Control plasmid (Plasmid lacking editing cassette and sgRNA) (Fig S5)	ampicillin resistance	
pKAW109	empty plasmid used for Cas9-assisted editing control experiments; ampicillin resistance; pMB1 origin of replication	this study

**Table S3.** PCR primers used in this work. F and R designate forward and reverse primers, respectively.		
Primers for amplification of the donor and guide plasmid backbones used in Cas9-assisted genome editing		
primer	sequence	use
Donor2_bbF	GTTTTAGAGCTAGAAATAGCAAGTTAAAATAAGGCTAGTCCGTTATCAAC	amplification of donor plasmid backbone from pAM041
Donor2_bbR	ACAGCTTCAAGTAGTCGGGGATGTC	
P623_RmvSpacerF	GTTTTAGAGCTAGAAATAGC	amplification of guide plasmid backbone from pAM041
P624_ RmvSpacerR	ACTAGTATTATACCTAGGAC	
Primer for Sanger sequencing donor and guide plasmid inserts		
primer	sequence	use
2nd_seqF	ATAAGGGCGACACGGAAATGTTGAATACTC	sequencing primer to verify insert in donor and guide plasmids
Primers for colony PCR and/or Sanger sequencing to confirm Cas9-assisted editing		
primer	sequence	target gene / amplifies region around indicated edited codon(s)
acs328-SeqF	GCATGTGGTGGTACTGAAGCGTACTG	*acs* / codon 328
acs328-SeqR	CTTCCGGGTTAATTGGCTCGCCCAC	*acs* / codon 328
acs610-SeqF	GGAACCGTCACCAGAACTGTACGCAGAAG	*acs* / codon 610
acs610-SeqR	CAAACCGTTACCGACTCGCATCGGGC	*acs* / codon 610
adhE490_seq_F	TCTCAGGGTGGTATCGGTGACCTG	*adhE* / codon 490
adhE490_seq_R	TGTTGTGGAAGCCGTTATAGTGCCTCAG	*adhE* / codon 490
cyaA499_seq_F	GCTGGAAGCCTATTCCTGGGAATAC	*cyaA* / codon 499
cyaA499_seq_R	CAGTTTCTGTACCGATACATTCAGGCG	*cyaA* / codon 499
fbaB238_seq_F	CTGTATGCCAGCGTGGAGCAGG	*fbaB* / codon 238
fbaB238_seq_R	CTGCACGGCGTTAATCAGTTTCACG	*fbaB* / codon 238
pgi352_seq_F	CGTTGGCGAGTTTGGTATTG	*pgi* / codon 352
pgi352_seq_R	GATGATCAGAGAGCGGGTTATG	*pgi* / codon 352
ptsI_3’F	GAGCTGGAGCAGGAAATCATAG	*ptsI* / codon 513
ptsI_3’R	GAGAATGCGTGGTTGGTTTC	
pykF_seq_F	GAACTTCTCTCATGGTGACTATGCAG	*pykF* / codons 85 and 466
pykF_seq_R	GATGCTTCCATCGGATTCATCTTAGATA	
Primers used for lambda Red recombineering		
primer	sequence	use
KW459F	CTCCCTGACCTGGTGGTTGC	amplification of *pdxB* editing cassette from pKAW086
KW460R	ACTGACGTTCAGCCAGCGTTTCAA	
KW438F	AAAATATGCCTTATGCCCGCGACTT	bind within *pdxB* coding sequence to check for presence of *pdxB* in edited strains
KW439R	GCGTTAAAACCCAGTTTACACAGCAATGAT	

KW440F	GCGTCAGTGAGGTCACTTTCGT	bind outside of *pdxB* coding sequence to check for correct insertion of *kanR*
KW441R	ATCCTGCACGGTGATTGTCTTACC	
Primers used for I-SceI-assisted editing		
primer	sequence	use
KW061F	CACTTGGTTCATGGTCCAGCTTA	amplification of *rpoS* editing cassette from pKAW066 that inserts a T at position 98 in *rpoS*
KW062R	CAGTACATCAACCAGTACGCCTA	
KW385F	TAGCACCGGAACCAGTTCAACAC	bind outside of *rpoS* coding sequence; used to confirm correct editing
KW384R	GTTGTCGGTAGCAGACGGTCTGA	
Primers used for pKAW109 construction		
primer	sequence	use
KW027F	CGCAGGAAAGAACATGTG	amplification of oriV and ampicillin resistance gene from pAM041
KW181R	CCAGAAATCATCCTTAGCGAA	

Table S4. gBlocks used to construct donor plasmids used for Cas9-assisted editing.

Lower-case italics, extensions to direct Gibson Assembly with vector backbone; upper-case black, editing cassette; green, J23119 constitutive promoter; blue, 20-nt gRNA spacer sequence; red and magenta, target and synonymous immunizing mutations in or near the PAM site, respectively. (Target codons are underlined.)	
GB_ackA105_2nd (for construction of pDY339)	*caaggcctacgtgaagcaccccgccgacatccccgactacttgaagctgt*GGCTTTAGGTGCAGGCGCCGCTCACAGCGAAGCGCTCAACTTTATCGTTAATACTATTCTGGCACAAAAACCAGAACTGTCTGCGCAGCTGACTGCTATCGGTCACCGTATCGTACACGGCGGCGAAAAGTATACCAGCTCCGTAGTGATAGATGAGTCTGTTATTCAGGGTATTAAGGATGCAGCTTCTTTTGCACCGCTGCACAACCCGGCTCACCTGATCGGTATCGAAGAAGCTCTGAAATCTTTCCCACAGCTGAAAGACAAAAACGTTGCTGTATTTGACACCGCGTTCCACCAGACTATGCCGGAAGAGTCTTACCTCTTTGACAGCTAGCTCAGTCCTAGGTATAATACTAGTCTGTCCCCTCAGTTCATGTA*gttttagagctagaaatagcaagttaaaataaggctagtccgttatcaac*
GB_cyaA499_2nd (for construction of pDY348)	*caaggcctacgtgaagcaccccgccgacatccccgactacttgaagctgt*CCCGATCTCTCGGAACCGAATCTGACCTTTATTTATGTGCCGCCGGGCCGGGCTAACCGTTCAGGTTGGTATCTGTATAACCGCGCGCCAAATATTGAGTCGATCATCAGCCATCAGCCGCTGGAATATAACCGTTACCTGAATAAACTGGTGGCGTGGGCATGGTTCAATGGCCTGCTGACCTCGCGCACCCGTTTGTATATTAAAGGTAACGGCATTGTCGATTTGCCTAAGTTGCAGGAGATGGTCGCCGACGTGTCGCACCATTTCCCGCTGCGCTTACCTGCACCGACACCGAAGGCGCTCTACAGCCCGTGTGAGTTGACAGCTAGCTCAGTCCTAGGTATAATACTAGTCTGTCCCCTCAGTTCATGTA*gttttagagctagaaatagcaagttaaaataaggctagtccgttatcaac*
GB_pykF085_2nd (for construction of pDY449)	*caaggcctacgtgaagcaccccgccgacatccccgactacttgaagctgt*GAACTTCTCTCATGGTGACTATGCAGAACACGGTCAGCGCATTCAGAATCTGCGCAACGTGATGAGCAAAACTGGTAAAACCGCCGCTATCCTGCTTGATACCAAAGGTCCGGAAATCCGCACCATGAAACTGGAAGGCGGTAACGACGTTTCTTTGAAGGCTGGTCAGACCTTTACTTTCACCACTGATAAATCTGTTATCGGCAACAGCGAAATGGTTGCGGTAACGTATGAAGGTTTCACTACTGACCTGTCTGTTGGCAACACCGTACTGGTTGACGATGGTCTGATCGGTATGGAAGTTACCGCCTTGACAGCTAGCTCAGTCCTAGGTATAATACTAGTTGAACGTTTGTCCGGCCTTT*gttttagagctagaaatagcaagttaaaataaggctagtccgttatcaac*
GB_pykF090_2nd (for construction of pDY450)	*caaggcctacgtgaagcaccccgccgacatccccgactacttgaagctgt*GAACTTCTCTCATGGTGACTATGCAGAACACGGTCAGCGCATTCAGAATCTGCGCAACGTGATGAGCAAAACTGGTAAAACCGCCGCTATCCTGCTTGATACCAAAGGTCCGGAAATCCGCACCATGAAACTGGAAGGCGGTAACGACGTGAGCCTGAAAGCTGGTCAGACGTTTACTTTCACCACTGATAAATCTGTTATCGGCAACAGCGAAATGGTTGCGGTAACGTATGAAGGTTTCACTACTGACCTGTCTGTTGGCAACACCGTACTGGTTGACGATGGTCTGATCGGTATGGAAGTTACCGCCATTGAAGGTAACTTGACAGCTAGCTCAGTCCTAGGTATAATACTAGTTGAACGTTTGTCCGGCCTTT*gttttagagctagaaatagcaagttaaaataaggctagtccgttatcaac*

GB_pykF466_2nd (for construction of pDY452)	*caaggcctacgtgaagcaccccgccgacatccccgactacttgaagctgt*CGAAAAAACGGCTC ATCAGTTGGTACTGAGCAAAGGCGTTGTGCCGCAGCTTGTTAAAGAG ATCACTTCTACTGATGATTTCTACCGTCTGGGTAAAGAACTGGCTCTGC AGAGCGGTCTGGCACACAAAGGTGACGTTGTAGTTATGGTGAGCGGT GCACTGGTACCGAGCGGCACTACTAACACCGCAAGCGTTCACGTCCT GTAATATTGCTTTTGTGAATTAATTTGTATATCGAAGCGCCCTGATGGGC GCTTTTTTTATTTAATCGATAACCAGAAGCAATAAAAAATCAAATCGGA TTTCACTATATAATCTCACTTTATCTAAGATGAATCCGATTTGACAGCTA GCTCAGTCCTAGGTATAATACTAGTAGCGGAGCTTTAGTTCCATC*gttttag agctagaaatagcaagttaaaataaggctagtccgttatcaac*
GB_pykF470_2nd (for construction of pDY453)	*caaggcctacgtgaagcaccccgccgacatccccgactacttgaagctgt*CGAAAAAACGGCTC ATCAGTTGGTACTGAGCAAAGGCGTTGTGCCGCAGCTTGTTAAAGAG ATCACTTCTACTGATGATTTCTACCGTCTGGGTAAAGAACTGGCTCTGC AGAGCGGTCTGGCACACAAAGGTGACGTTGTAGTTATGGTGAGCGGT GCACTGGTACCGAGCGGCACTACTAACACCGCATCTGTTCACGTCCTG TAATATTGCTTTTGTGAATTAATTTGTATATCGAAGCGCCCTGATGGGCG CTTTTTTTATTTAATCGATAACCAGAAGCAATAAAAAATCAAATCGGAT TTCACTATATAATCTCACTTTATCTAAGATGAATCCGATTTGACAGCTAG CTCAGTCCTAGGTATAATACTAGTAGCGGAGCTTTAGTTCCATC*gttttagag ctagaaatagcaagttaaaataaggctagtccgttatcaac*

**Table S5**. 120-nt ssDNA editing cassettes used for Cas9-assisted editing and the associated spacer sequences used to direct Cas9 genome cleavage. Red and magenta, target and immunizing mutations, respectively. Target codons are underlined.		
target gene/codon	sequence	associated spacer sequence
acs / 328	GTGACCGGACACAGTTACTTGCTGTACGGCCCGCTGGCCTG CGGTGCGACCACGCTCATGTTCGAGGGCGTACCCAACTGG CCGACGCCTGCCCGTATGGCGCAGGTGGTGGACAAGCAT	CTGTCCCCTC AGTTCATGTA
acs / 328	GTGACCGGACACAGTTACTTGCTGTACGGCCCGCTGGCCTG CGGTGCGACCACGCTCATGTTTGAAGGCGTACCCAACTGGC CGACGCCTGCCCGTATGGCGCAGGTGGTGGACAAGCAT	CTGTCCCCTC AGTTCATGTA
acs / 328	GTGACCGGACACAGTTACTTGCTGTACGGCCCGCTGGCCTG CGGTGCGACCACGCTGATGTTCGAAGGCGTACCCAACTGG CCGACGCCTGCCCGTATGGCGCAGGTGGTGGACAAGCAT	CTGTCCCCTC AGTTCATGTA
acs / 328	GTGACCGGACACAGTTACTTGCTGTACGGCCCGCTGGCCTG CGGTGCGACCACGCTGATGTTTGAGGGCGTACCCAACTGG CCGACGCCTGCCCGTATGGCGCAGGTGGTGGACAAGCAT	CTGTCCCCTC AGTTCATGTA
acs / 610	GACGTGCTGCACTGGACCGACTCCCTGCCTAAAACCCGCTC CGGCAAAATCATGCGCCGTATTCTGCGTAAGATTGCGGCGG GCGATACCAGCAACCTGGGCGATACCTCGACGCTTGCC	CTGTCCCCTC AGTTCATGTA
acs / 619	TGGACCGACTCCCTGCCTAAAACCCGCTCCGGCAAAATTAT GCGCCGTATTCTGCGTAAGATTGCAGCGGGCGATACCAGCA ACCTGGGCGATACCTCGACGCTTGCCGATCCTGGCGTA	CTGTCCCCTC AGTTCATGTA
acs / 624	ACTCCCTGCCTAAAACCCGCTCCGGCAAAATTATGCGCCGT ATTCTGCGTAAGATTGCGGCGGGCGATACCTCGAACCTGGG CGATACCTCGACGCTTGCCGATCCTGGCGTAGTCGAG	CTGTCCCCTC AGTTCATGTA
acs / 610	GACGTGCTGCACTGGACCGACTCCCTGCCTAAAACCCGCTC CGGCAAAATCATGCGCCGTATTCTGCGCAAAATTGCGGCGG GCGATACCAGCAACCTGGGCGATACCTCGACGCTTGCC	CTGTCCCCTC AGTTCATGTA
acs / 610	GACGTGCTGCACTGGACCGACTCCCTGCCTAAAACCCGCTC CGGCAAAATTATGCGCCGTATTCTGCGTAAAATTGCGGCGG GCGATACCAGCAACCTGGGCGATACCTCGACGCTTGCC	CTGTCCCCTC AGTTCATGTA
acs / 610	GACGTGCTGCACTGGACCGACTCCCTGCCTAAAACCCGCTC CGGCAAAATTATGCGCCGTATTCTGCGCAAGATTGCGGCGG GCGATACCAGCAACCTGGGCGATACCTCGACGCTTGCC	CTGTCCCCTC AGTTCATGTA
pgi / 352	CACAACGTTACCGTTACGGTCAACATACTTACCGTTGGACT CCATATTCCCCTGCTGGAAGTACGCCGCAAACCGGTGCATA TACTGGTCATACGGCAGAATCGCTTCAGTTTCCGCACC	TTTGCAGCCT ATTTTCAACA
pgi / 352	CACAACGTTACCGTTACGGTCAACATACTTACCGTTGGACT CCATATTGCCCTGCTGGAAGTACGCCGCGAACCGGTGCATA TACTGGTCATACGGCAGAATCGCTTCAGTTTCCGCACC	TTTGCAGCCT ATTTTCAACA
pgi / 352	CACAACGTTACCGTTACGGTCAACATACTTACCGTTGGACT CCATATTGCCCTGCTGGAAGTACGCCGCAAAACGGTGCATA TACTGGTCATACGGCAGAATCGCTTCAGTTTCCGCACC	TTTGCAGCCT ATTTTCAACA
pgi / 352	CACAACGTTACCGTTACGGTCAACATACTTACCGTTGGACT CCATATTCCCCTGCTGGAAGTACGCCGCGAAACGGTGCATA TACTGGTCATACGGCAGAATCGCTTCAGTTTCCGCACC	TTTGCAGCCT ATTTTCAACA
fbaB / 238	TGACCGGTCAGGCAAACCATCTGGCGGCAACCATCGGTGC AGATATCGTCAAACAAAAAATGGCCGAGAATAACGGCGGC TATAAAGCAATTAATTACGGTTACACCGACGATCGTGTTT	TCTGGCGGCA ACAATAGGAG

adhE / 486	CGCTGGATGAAGTGATTACTGATGGCCACAAACGTGCGCTC ATCGTGACCGACCGGTTTCTGTTCAACAATGGTTATGCTGAT CAGATCACTTCCGTACTGAAAGCAGCAGGCGTT	CTGTCCCCTC AGTTCATGTA
adhE / 490	CGCTGGATGAAGTGATTACTGATGGCCACAAACGTGCGCTC ATCGTGACTGACCGGTTTTTATTCAACAATGGTTATGCTGAT CAGATCACTTCCGTACTGAAAGCAGCAGGCGTTGAAA	CTGTCCCCTC AGTTCATGTA
adhE / 496	AAGTGATTACTGATGGCCACAAACGTGCGCTCATCGTGACT GACCGGTTTCTGTTCAACAATGGTTATGCTGATCAGATCACT TCCGTACTGAAAGCAGCAGGCGTTGAAACTGAAGTCT	CTGTCCCCTC AGTTCATGTA
adhE / 496	AAGTGATTACTGATGGCCACAAACGTGCGCTCATCGTGACT GACCGGTTTCTGTTCAACAATGGTTATGCCGATCAGATCACT TCCGTACTGAAAGCAGCAGGCGTTGAAACTGAAGTCT	CTGTCCCCTC AGTTCATGTA
adhE / 496	AAGTGATTACTGATGGCCACAAACGTGCGCTCATCGTGACT GACCGGTTTCTGTTCAACAATGGTTATGCGGATCAGATCAC TTCCGTACTGAAAGCAGCAGGCGTTGAAACTGAAGTCT	CTGTCCCCTC AGTTCATGTA

**Table S6.** Lambda Red recombineering editing cassette used to replace *pdxB* with *kanR*
Red, homology arm (HA) upstream of *pdxB*; black, sequences containing promoters, terminators and FRT sites; grey, *kanR*; cyan, HA downstream of *pdxB*.
CTCCCTGACCTGGTGGTTGCCCAGGAGGAGGGCCGGAAATAGGTTGTATCATTACGTATC<br>CTTATACCTGAAATCTTCGCAAGTATGCCTGGCCGCGAGATTATGGCACACTTGTCCGGTT<br>AACTCTCGTCTCATACAGGTAACACAAACGTGATTCCGGGGATCCGTCGACCTGCAGTTCG<br>AAGTTCCTATTCTCTAGAAAGTATAGGAACTTCAGAGCGCTTTTGAAGCTCACGCTGCCGC<br>AAGCACTCAGGGCGCAAGGGCTGCTAAAGGAAGCGGAACACGTAGAAAGCCAGTCCGCA<br>GAAACGGTGCTGACCCCGGATGAATGTCAGCTACTGGGCTATCTGGACAAGGGAAAACGC<br>AAGCGCAAAGAGAAAGCAGGTAGCTTGCAGTGGGCTTACATGGCGATAGCTAGACTGGG<br>CGGTTTTATGGACAGCAAGCGAACCGGAATTGCCAGCTGGGGCGCCCTCTGGTAAGGTTG<br>GGAAGCCCTGCAAAGTAAACTGGATGGCTTTCTTGCCGCCAAGGATCTGATGGCGCAGGG<br>GATCAAGATCTGATCAAGAGACAGGATGAGGATCGTTTCGCATGATTGAACAAGATGGAT<br>TGCACGCAGGTTCTCCGGCCGCTTGGGTGGAGAGGCTATTCGGCTATGACTGGGCACAAC<br>AGACAATCGGCTGCTCTGATGCCGCCGTGTTCCGGCTGTCAGCGCAGGGGCGCCCGGTTCT<br>TTTTGTCAAGACCGACCTGTCCGGTGCCCTGAATGAACTGCAGGACGAGGCAACGCGGCT<br>ATCGTGGCTGGCCACGACGGGCGTTCCTTGCGCAGCTGTGCTCGACGTTGTCACTGAAGCG<br>GGAAGGGACTGGCTGCTATTGGGCGAAGTGCCGGGGCAGGATCTCCTGTCATCTCACCTT<br>GCTCCTGCCGAGAAAGTATCCATCATGGCTGATGCAATGCGGCGGCTGCATACGCTTGAT<br>CCGGCTACCTGCCCATTCGACCACCAAGCGAAACATCGCATCGAGCGAGCACGTACTCGG<br>ATGGAAGCCGGTCTTGTCGATCAGGATGATCTGGACGAAGAGCATCAGGGGCTCGCGCCA<br>GCCGAACTGTTCGCCAGGCTCAAGGCGCGCATGCCCGACGGCGAGGATCTCGTCGTGACC<br>CATGGCGATGCCTGCTTGCCGAATATCATGGTGGAAAATGGCCGCTTTTCTGGATTCATCG<br>ACTGTGGCCGGCTGGGTGTGGCGGACCGCTATCAGGACATAGCGTTGGCTACCCGTGATA<br>TTGCTGAAGAGCTTGGCGGCGAATGGGCTGACCGCTTCCTCGTGCTTTACGGTATCGCCGC<br>TCCCGATTCGCAGCGCATCGCCTTCTATCGCCTTCTTGACGAGTTCTTCTAATAAGGGGAT<br>CTTGAAGTTCCTATTCCGAAGTTCCTATTCTCTAGAAAGTATAGGAACTTCGAAGCAGCTC<br>CAGCCTACAGTGCATCATCCGGCACGTTAATCTCTTCTTCATGCTCTCTGCTGTAACATTGG<br>CAGGGAGCTTTGCTATTTCTGGAGTAAACCACCATGTCTGAAGGCTGGAACATTGCCGTCC<br>TGGGCGCAACTGGCGCTGTGGGCGAAGCCCTGCTTGAAACGCTGGCTGAACGTCAGT

**Table S7.** Editing cassette used to insert T at position 98 in *rpoS* with I-SceI-assisted editing
Red, homology arm (HA) upstream of the edit; yellow, edit; cyan, downstream HA; black, segment containing terminators; green, segment containing an I-SceI cut-site; black, segment containing a promoter; grey, streptomycin resistance gene; red, short HA upstream of the edit; yellow, edit; blue, downstream HA.
CACTTGGTTCATGGTCCAGCTTATGGGACAACTCACGTGCGGTTCGCAGGTAAACGTTCAG<br>CTCCTTTACGATGTGAATCGGCAAACGAATAGTACGGGTTTGGTTCATAATCGCCCGTTCA<br>ATCGTCTGGCGAATCCACCAGGTTGCGTATGTTGAGAAGCGGAAACCACGTTCCGGGTCA<br>AACTTCTCTACCGCGCGGATCAGCCCCAGGTTGCCCTCTTCGATAAGGTCCAGCAACGCCA<br>GACCACGATTGCCATAACGGCGGGCAATTTTTACCACCAGACGCAAGTTACTCTCGATCAT<br>CCGGCGGCGAGAGGCGACATCTCCACGCAGTGCGCGACGCGCAAAATAAACTTCTTCTTC<br>GGCCGTTAACAGTGGTGAATAACCAATCTCACCAAGGTAAAGCTGAGTCGCGTCCAACAC<br>ACGCTGTGTGGCTCCCTGCGATAACAGTTCCTCTTCGGCCAAATCGTTATCACTGGGTTCC<br>TTGTTCTACTAAGGCCATCTCAAGAGTGGCAGCGGTTCTGTTAAGTAACTGAACCCAATGT<br>CGTTAGTGACGCTTACCCGCAAAAAACCCCGCTTCGGCGGGGTTTTTCGCTCTTAAGAGG<br>TCACTGACCTAACAAAAAAAAAACCCCGCCCCTGACAGGGCGGGGTTTTTTTTGGTCTTGA<br>GTGGCAGAGTCAGTTATCGCGAGCAGTATGTAAGTAGATCCTCAGTGTCAGCTAGGGATA<br>ACAGGGTAATCCTGGTGTCCCTGTTGATACCGGGAAGCCCTGGGCCAACTTTTGGCGAAA<br>ATGAGACGTTGATCGGCACGTAAGAGGTTCCAACTTTCACCATAATGAAATAAGATCACT<br>ACCGGGCGTATTTTTTGAGTTATCGAGATTTTCAGGAGCTAAGGAAGCTAAAATGAGGGA<br>AGCGGTGATCGCCGAAGTATCGACTCAACTATCAGAGGTAGTTGGCGTCATCGAGCGCCA<br>TCTCGAACCGACGTTGCTGGCCGTACATTTGTACGGCTCCGCAGTGGATGGCGGCCTGAA<br>GCCACACAGTGATATTGATTTGCTGGTTACGGTGACCGTAAGGCTTGATGAAACAACGCG<br>GCGAGCTTTGATCAACGACCTTTTGGAAACTTCGGCTTCCCCTGGAGAGAGCGAGATTCTC<br>CGCGCTGTAGAAGTCACCATTGTTGTGCACGACGACATCATTCCGTGGCGTTATCCAGCTA<br>AGCGCGAACTGCAATTTGGAGAATGGCAGCGCAATGACATTCTTGCAGGTATCTTCGAGC<br>CAGCCACGATCGACATTGATCTGGCTATCTTGCTGACAAAAGCAAGAGAACATAGCGTTG<br>CCTTGGTAGGTCCAGCGGCGGAGGAACTCTTTGATCCGGTTCCTGAACAGGATCTATTTGA<br>GGCGCTAAATGAAACCTTAACGCTATGGAACTCGCCGCCCGACTGGGCTGGCGATGAGCG<br>AAATGTAGTGCTTACGTTGTCCCGCATTTGGTACAGCGCAGTAACCGGCAAAATCGCGCC<br>GAAGGATGTCGCTGCCGACTGGGCAATGGAGCGCCTGCCGGCCCAGTATCAGCCCGTCAT<br>ACTTGAAGCTAGACAGGCTTATCTTGGACAAGAAGAAGATCGCTTGGCCTCGCGCGCAGA<br>TCAGTTGGAAGAATTTGTCCACTACGTGAAAGGCGAGATCACCAAGGTAGTCGGCAAATA<br>ATTATCACTGGGTTCCTTGTTCTACTAAGGCCTTTTCGTCAAAAACCTCAACTCCGTTCTCA<br>TCAAATTCCGCATCTTCATTTAAATCATGAACTTTCAGCGTATTCTGACTCATAAGGTGGC<br>TCCTACCCGTGATCCCTTGACGGAACATTCAAGCAAAAGCCTGGTTCCGCCGATTTATCGC<br>TGCGGCAAATAACGCAGCGGGTTTACGGATTTCCCCTTGTAACGAATTTCAAAATGCAAG<br>CGTGTTGAACTGGTTCCGGTGCTACCCATGGTCGCTATTTTTTGCCCCGCCTTAACTTCTTG<br>TTGTTCCCGGACCAGCATTGTGTCGTTATGGGCGTAGGCACTCAGGTAATCATCATTATGT<br>TTGATGATAATCAGATTACCGTAGCCGCGCAGCGCGTTACCAGCATAAACAACGCGGCCA<br>TCTGCGGTCGCGATAATTGCCTGTCCTTTGCTGCCTGCGATATCAATCCCCTTGTTGCCCCC<br>CTCAGAAGCGCCAAAGGTTTCGATCACTTTGCCCTCAGTCGGCCAGCGCCAGGTGGAGAT<br>AGGCGTACTGGTTGATGTACTG

**Table S8.** 60-nt oligonucleotides containing spacer sequences used to make guide plasmids		
Uppercase, 20-nt spacer; lowercase, sequences that overlap the guide plasmid backbone. The complementary 60-nt oligonucleotide fragments were annealed and then ligated into the pAM041 backbone by Gibson assembly.		
primer	sequence	guide plasmid constructed
mCherry_sgRNA_F	gtcctaggtataatactagtCTGTCCCCTCAGTTCATGTAgt tttagagctagaaatagc	pDY320
mCherry_sgRNA_R	gctatttctagctctaaaacTACATGAACTGAGGGGACAG actagtattatacctaggac	pDY320
pgi352_2nd_sgRNA_F	gtcctaggtataatactagtTTTGCAGCCTATTTTCAACAgt tttagagctagaaatagc	pDY332
pgi352_2nd_sgRNA_R	gctatttctagctctaaaacTGTTGAAAATAGGCTGCAAAa ctagtattatacctaggac	pDY332
fbaB_2nd_sgRNA_F	gtcctaggtataatactagtTCTGGCGGCAACAATAGGAG gttttagagctagaaatagc	pDY334
fbaB_2nd_sgRNA_R	gctatttctagctctaaaacCTCCTATTGTTGCCGCCAGAa ctagtattatacctaggac	pDY334
ptsI_2nd_sgRNA_F	gtcctaggtataatactagtAGAGCAACCTTGTTACTATTg ttttagagctagaaatagc	pDY337
ptsI_2nd_sgRNA_R	gctatttctagctctaaaacAATAGTAACAAGGTTGCTCTa ctagtattatacctaggac	pDY337

Supplemental References

1. Bassalo, M.C., Garst, A.D., Halweg-Edwards, A.L., Grau, W.C., Domaille, D.W., Mutalik, V.K., Arkin, A.P. and Gill, R.T. (2016) Rapid and efficient one-step metabolic pathway integration in *E. coli*. *ACS Synth. Biol.*, **5**, 561–568.

2. Dunn, A.K., Martin, M.O. and Stabb, E.V. (2005) Characterization of pES213, a small mobilizable plasmid from *Vibrio fischeri*. *Plasmid*, **54**, 114–134.

3. Yang, D.-D., Rusch, L.M., Widney, K.A., Morgenthaler, A.B. and Copley, S.D. (2024) Synonymous edits in the *Escherichia coli* genome have substantial and condition-dependent effects on fitness. *PNAS*, **121**, e2316834121.
